# Resolution of Discoid Lupus Alopecia With Systemic Hydroxychloroquine and Topical Pimecrolimus Combination Therapy

**DOI:** 10.7759/cureus.63419

**Published:** 2024-06-28

**Authors:** Kristine R Liang, Cynthia Lee, Alexis Hilts, H.L. Greenberg

**Affiliations:** 1 School of Medicine, Kirk Kerkorian School of Medicine at University of Nevada Las Vegas, Las Vegas, USA; 2 Transitional Year Residency Program, Sunrise Health Graduate Medical Education (GME) Consortium, HCA Healthcare, Las Vegas, USA; 3 Dermatology, Las Vegas Dermatology, Las Vegas, USA

**Keywords:** cutaneous lupus erythematous, lupus, hydroxychloroquine, pimecrolimus, discoid alopecia, combination therapy, discoid lupus erythematosus (dle)

## Abstract

Discoid lupus erythematosus (DLE) is an autoimmune skin condition that is typically part of the cutaneous manifestation of systemic lupus erythematosus (SLE). DLE is characterized by erythematous patches that can progress to depigmentation and alopecia, leading to scarring and permanent hair loss if left untreated. Herein, we present a unique case of localized DLE on the scalp in a 46-year-old female with no prior history of autoimmune disorders. The patient underwent several medication trials, including intralesional corticosteroids, topical calcineurin inhibitors, topical corticosteroids, and systemic hydroxychloroquine, with limited success in treating her discoid alopecia. Subsequently, a combination therapy of oral hydroxychloroquine and topical pimecrolimus significantly improved her scalp lesion. This case highlights the efficacy of combination therapy in managing localized DLE, providing valuable insights for future research focused on DLE alopecia management and optimizing treatment strategies for similar cases.

## Introduction

Systemic lupus erythematosus (SLE) is an autoimmune disorder that can affect several areas of the body, including the skin, with cutaneous manifestations present in more than half of those affected [1]. Chronic cutaneous lupus erythematosus (CLE) can manifest in various forms, including localized and generalized discoid lupus erythematosus (DLE), with generalized DLE being less common [2]. Currently, there is a lack of literature examining non-autoimmune causes or risk factors of DLE. Localized discoid lupus lesions are typically triggered by ultraviolet light and found on sun-exposed areas above the neck, including the ears, bridge of the nose, and scalp [2]. When found on the scalp, these lesions can first appear as erythematous patches and progress to white patches devoid of hair [2]. Histologic examination typically demonstrates hyperkeratosis, prominent follicular plugging, thickening of the basement membrane, and thinning epidermis [2, 3]. DLE can progress to irreversible scarring alopecia, which is more common in patients who experience chronic disease and a younger age of onset [3]. Early diagnosis and treatment of DLE are imperative to prevent skin atrophy and scarring [1]. We discuss the clinical and histological findings of a unique case of localized scarring DLE in a 46-year-old woman without a history of autoimmune disorders. This case was previously presented as a poster at the 2022 Nevada Society for Dermatology and Dermatologic Surgery (NSDDS) Annual Meeting.

## Case presentation

**Figure 1:**
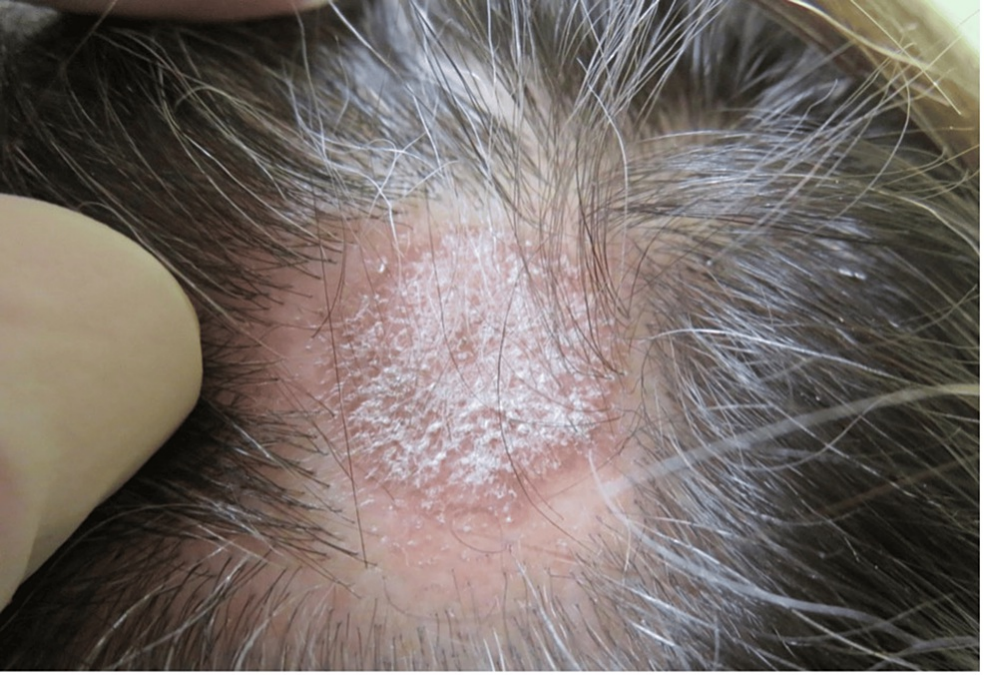
Initial presentation of the patient’s scalp with an area of alopecia

**Figure 2:**
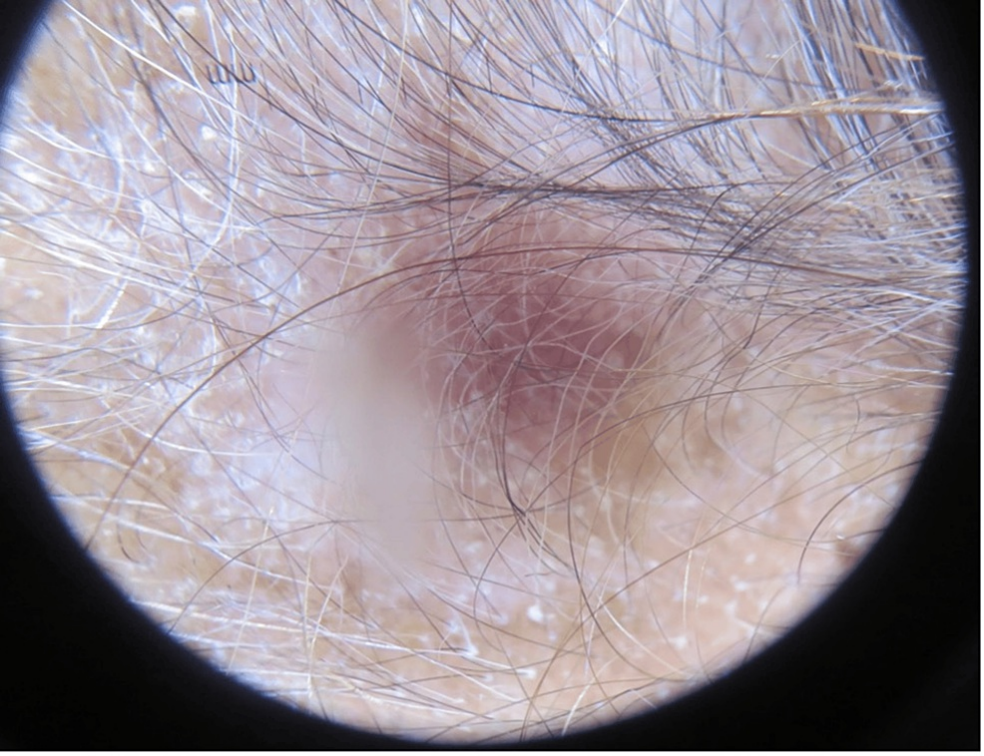
Dermoscopic examination of patient’s scalp showing white scales, follicular keratotic plugs, and perifollicular scaling

**Figure 3:**
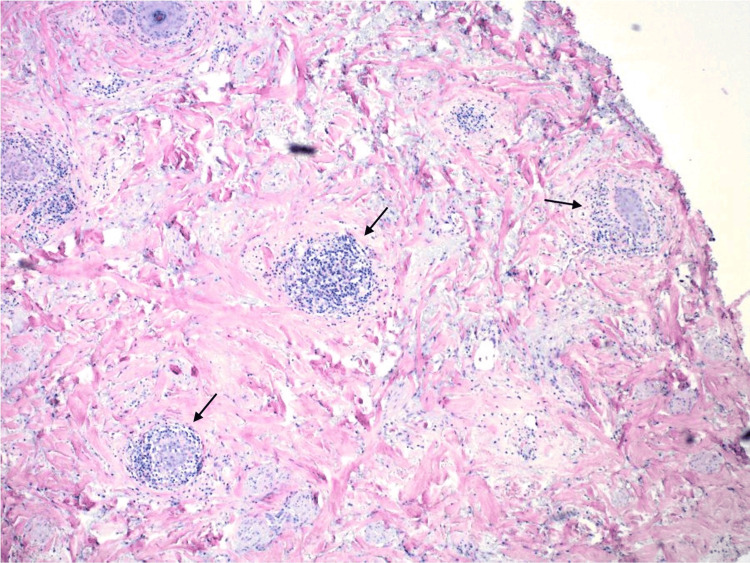
Horizontal section of scalp biopsy demonstrating perifollicular lymphocytic infiltrates (black arrows)

A 46-year-old Caucasian female with no significant medical history presented to the clinic with a chief complaint of hair loss on her scalp for six months. Upon examination, there was an area of scarring alopecia localized to the frontal scalp (Figure 1). Initially, it was suspected to be neurotic excoriations due to the increased level of situational stress reported by the patient. However, dermoscopic examination revealed white scales, follicular keratotic plugs, and perifollicular scaling (Figure 2), which are findings more consistent with DLE. A biopsy of the lesion was performed and demonstrated pathological findings of hyperkeratosis, follicular plugging, epidermal atrophy, liquefactive degeneration of the basal cell layer with an underlying thickened basement membrane, a superficial and deep perivascular and periadnexal lymphohistiocytic infiltrate, and perifollicular lymphocytic infiltrates (Figure 3). These pathological features are also consistent with a diagnosis of DLE.

**Figure 4:**
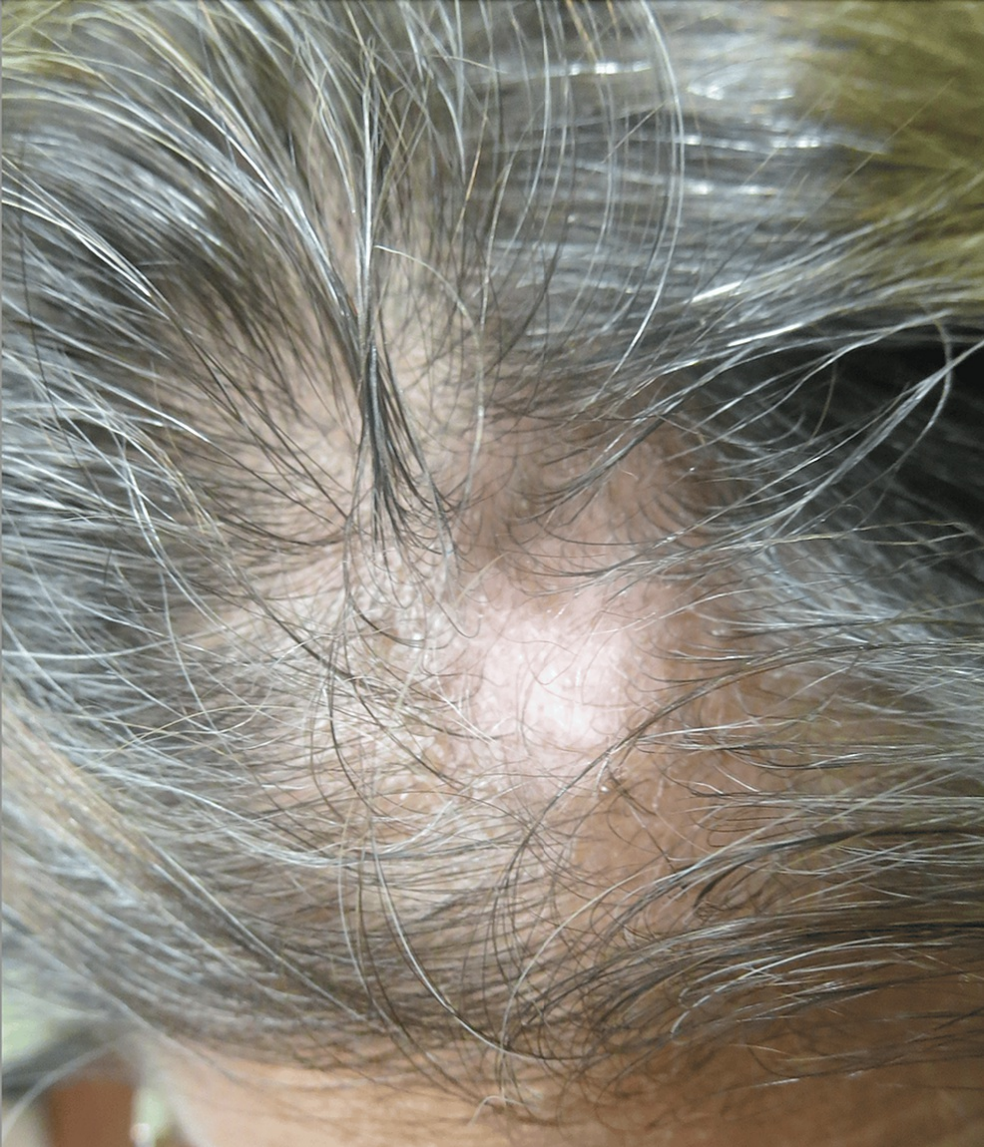
Improvement of scarring alopecia of the frontal scalp following eight weeks of PO hydroxychloroquine 200 mg twice daily and 1% pimecrolimus cream

**Figure 5:**
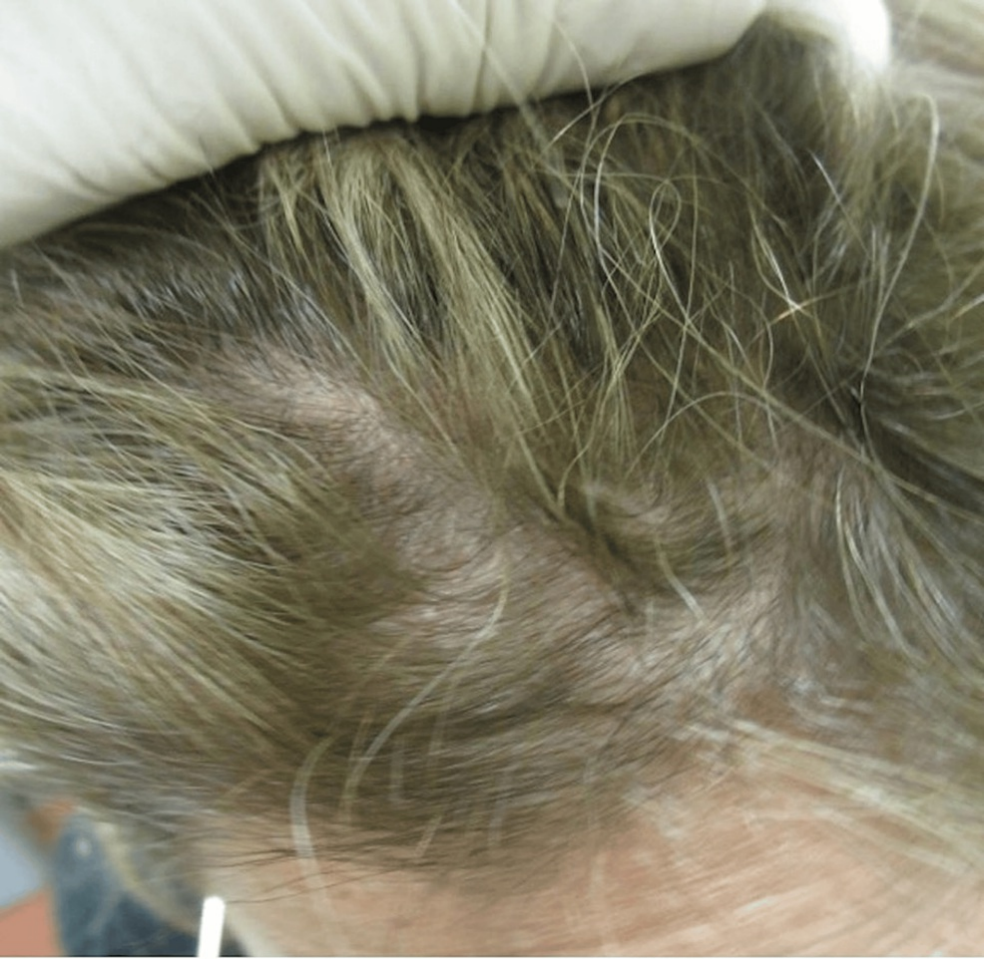
Complete resolution of discoid alopecia and hair regrowth one year after treatment

The patient was given a 2.5 mg/cc triamcinolone injection and was prescribed 0.1% tacrolimus ointment. However, due to the patient’s complaint of a burning sensation on the scalp, topical tacrolimus was discontinued. Treatment with 0.05% clobetasol ointment was started, but unfortunately, it did not improve the area of alopecia after one month of use. Subsequently, the patient was initiated on eight weeks of oral hydroxychloroquine 200 mg twice daily in combination with topical 1% pimecrolimus cream. After eight weeks of combination therapy, the patient experienced significant improvement (Figure 4). At her one-year follow-up, her scalp demonstrated hair regrowth and long-term resolution of her scarring alopecia (Figure 5).

## Discussion

The pathophysiology of CLE results from an interplay between genetic predisposition and environmental factors such as sunlight, cigarette smoke, trauma (Koebner phenomenon), and infections. The subtype, DLE, has a female predominance typically in the fourth and fifth decades of life, with a four-fold higher prevalence in African-American individuals compared to Caucasians [4]. Notably, our case involves a rare occurrence of spontaneous, localized DLE alopecia in a Caucasian female without a history of autoimmune disorders. The precise prevalence of spontaneous DLE in the current literature remains unclear. There is also a paucity of data comparing the effective duration of combined systemic therapy with topical agents.

Treatment for both CLE and DLE shares similar principles to manage symptoms and prevent flare-ups. The specific treatment plan may vary depending on the severity of symptoms and individual response to medications. When diagnosed early, first-line treatments for DLE alopecia include oral antimalarials such as hydroxychloroquine, and topical or intralesional corticosteroids [5]. Topical calcineurin inhibitors are alternative first-line treatments for patients who cannot tolerate corticosteroids. Additional options include methotrexate, topical and systemic retinoids, dapsone, thalidomide, and mycophenolate mofetil; however, the side effect profile of these medications may be unfavorable for some patients. Refractory cases have been shown to benefit from rituximab, intravenous immunoglobulin (IVIG), and ustekinumab, with the use of other biologics currently being investigated [5]. 

Our patient was treated with a triamcinolone injection, an intralesional corticosteroid, and tacrolimus ointment, a topical calcineurin inhibitor. Due to a reported burning sensation on the scalp, tacrolimus was replaced with clobetasol ointment, a topical corticosteroid. However, despite a month-long treatment with clobetasol, no clinical improvement was noted. She was then started on oral hydroxychloroquine, an antimalarial agent, with pimecrolimus ointment, an alternative topical calcineurin inhibitor. After eight weeks of treatment, her scalp showed significant improvement.

The most effective pharmacologic therapy for CLE was analyzed in a systematic review by Hannon et al., where the authors found that, compared to placebo, hydroxychloroquine and methotrexate demonstrated similar efficacy in treating the primary lesions of CLE over a 12-month period. Additionally, hydroxychloroquine was associated with fewer recurrent flares at the six-month post-treatment mark [6]. In another systematic review conducted by Jessop et al., the authors found agreeable evidence that hydroxychloroquine 400 mg daily was an effective therapy for CLE and was comparable to acitretin 50 mg in terms of complete resolution of skin lesions. Adverse effects were associated with acitretin in four participants, resulting in the cessation of treatment due to dry lips and gastrointestinal upset [7]. In a single study of 78 participants using topical steroid monotherapy, fluocinonide cream 0.05% was three times as effective as hydrocortisone 1% (27% versus 10%). Monotherapy with calcineurin inhibitors, such as tacrolimus 0.1% and pimecrolimus 1%, did not show significant clearance of cutaneous SLE or DLE [7].

In addition to systemic manifestations of DLE, including musculoskeletal symptoms, photosensitivity, and hematological sequelae [8], DLE can be cosmetically disfiguring. Presentation on frequently exposed skin, such as the face, upper extremities, and scalp, with or without subsequent alopecia, can have profound impacts on quality of life. Our patient experienced similar cosmetic issues such as alopecia, scalp dyspigmentation, and atrophy. Patients with cosmetic disfigurement are at risk for anxiety, depression, and low self-esteem [9]. A study measuring disease progression and quality of life in patients with cutaneous lupus found that improvement in disease did not directly correlate with improvement in quality of life. These findings suggest residual scarring and visible disfigurement are significant factors and should be considered in the long-term regimen for cutaneous lupus [10]. Within a two-month timeline, we successfully resolved our patient’s alopecia, scalp dyspigmentation, and atrophy, resulting in hair regrowth and ensuring her quality of life remained intact to the fullest extent.

## Conclusions

We present a case of complete resolution of DLE alopecia following a two-month regimen of PO hydroxychloroquine and topical pimecrolimus. DLE is well-known for its potential to cause permanent alopecia due to follicle destruction. Cosmetic disfigurement from the sequelae of DLE can cause great psychological distress in affected patients. Existing literature seems to favor hydroxychloroquine monotherapy; however, there remains a gap in standardizing the sufficient duration of combination therapy. Our case demonstrates promising benefits achieved within a reasonable timeline, favoring combination treatment with oral hydroxychloroquine and topical pimecrolimus. This finding is useful for future cases focused on DLE alopecia management.
